# Biological Effects of C_60_ Fullerene Revealed with Bacterial Biosensor—Toxic or Rather Antioxidant?

**DOI:** 10.3390/bios9020081

**Published:** 2019-06-21

**Authors:** Sergey Emelyantsev, Evgeniya Prazdnova, Vladimir Chistyakov, Igor Alperovich

**Affiliations:** Academy of Biology and Biotechnologies, Southern Federal University, 344090 Rostov-on-Don, Russia; emelyancev@sfedu.ru (S.E.); prazdnova@sfedu.ru (E.P.); vladimirchi@sfedu.ru (V.C.)

**Keywords:** fullerene C_60_, antioxidant activity, toxicity, oxidative stress, reactive oxygen species, hydrogen peroxide, *Escherichia coli*, biosensors, bioluminescence assay

## Abstract

Nanoparticles have been attracting growing interest for both their antioxidant and toxic effects. Their exact action on cells strongly depends on many factors, including experimental conditions, preparation, and solvents used, which have contributed to the confusion regarding their safety and possible health benefits. In order to clarify the biological effects of the most abundant fullerene C_60_, its impact on the *Escherichia coli* model has been studied. The main question was if C_60_ would have any antioxidant influence on the cell and, if yes, whether and to which extent it would be concentration-dependent. An oxidative stress induced by adding hydrogen peroxide was measured with an *E. coli* MG1655 pKatG-lux strain sensor, with its time evolution being recorded in the presence of fullerene C_60_ suspensions of different concentrations. Optimal conditions for the fullerene C_60_ solubilization in TWEEN 80 2% aqueous solution, together with resulting aggregate sizes, were determined. Results obtained for the bacterial model can be extrapolated on eukaryote mitochondria. The ability of C_60_ to penetrate through biological membranes, conduct protons, and interact with free radicals is likely responsible for its protective effect detected for *E. coli*. Thus, fullerene can be considered as a mitochondria-targeted antioxidant, worth further researching as a prospective component of novel medications.

## 1. Introduction

Fullerenes comprise a separate class of carbon allotropes with many synthesized modifications. The most stable and common fullerene among them is buckminsterfullerene C_60_, named after architect Buckminster Fuller, who invented geodesic domes resembling the C_60_ molecule. It has a spheroid structure, with carbon atoms being located at the vertices of 12 pentagons and 20 hexagons. A hypothetical, highly symmetrical carbon molecule, akin to a soccer ball, was initially predicted back in 1970 by Japanese scientist E. Osawa [1]. It was not until 1985 that H. Kroto, J. R. Heath, S. O’Brien, Robert Curl, and R. Smalley discovered fullerenes by examining the mass spectra of graphite vapor induced by laser irradiation and spotting peaks corresponding to molecule clusters of 60 and 70 atoms [2].

As buckminsterfullerene became an object of intensive research, its safety was questioned, so it still inspires scientific disputes. Many authors proposed the toxicity of unmodified fullerene C_60_ [3,4,5], which contradicts earlier findings [6,7] on the non-toxicity of C_60_. Interestingly, one of the proponents of fullerene toxicity later acknowledged the methodological drawbacks of the first experiments, namely, the presence of a sufficiently large concentration of tetrahydrofuran in the samples [8], which led to the biased conclusion about fullerene being toxic.

The paper [9] emphasizes that assessing safety of nanomaterials should take into account not only their intrinsic toxicity, but also interaction with other compounds and properties of nanoparticles in aqueous solutions. Thus, the matter of fullerene toxicity goes far beyond C_60_ intrinsic properties.

According to the paper [10], the crystalline form of C_60_, namely, fullerene films of density of 10, 20, 30 μg/cm^2^, did not show any toxic effect on the cell cultures of the MA-104 line (renal epithelium of the green monkey) without intensive illumination, while exhibiting a powerful phototoxic effect when irradiated with a halogen lamp. The water-soluble complex C_60_/PVP (polyvinylpyrrolidone) was not only shown to be non-toxic, but also attenuated the harmful effect of PVP itself [11]. Authors of the latter research concluded that the manifestation of the fullerene toxic effect depends on the method of its dissolution, used surfactants and/or modifiers, the degree of dispersion in solution, and conditions of the biological experiment; in particular, a prooxidant effect was found on UV light and an antioxidant one without UV illumination.

Thanks to its structure, fullerene C_60_ can bind up to six electrons [12,13], which move rapidly around the fullerene cage due to dipole moments. When C_60_ is exposed to light, electrons are pushed to a higher energy level, resulting in an excited singlet C_60_ state reacting with an oxygen molecule (O_2_) to form singlet oxygen (^1^O_2_), i.e., oxygen with a free electron in the outer orbit. Fullerenes are very efficacious generators of singlet oxygen with a quantum yield of ^1^O_2_ close to one. They absorb light efficiently in the ultraviolet and moderately in the visible regions of the spectrum [14], which paves the way for their application in photodynamic therapy [15].

Biological activity of C_60_ is complex and can be explained by several factors such as: (1) lipophilicity that determines membranotropic properties, (2) electron deficiency, which makes interaction with free radicals possible, (3) and the ability of fullerene in excited state to transfer energy to the oxygen molecule and convert it into singlet oxygen [10]. More detailed literature review on the topic is available, e.g., in [16].

In 2013, we hypothesized the ability of fullerene to be a proton carrier [12]. Fullerene was speculated to be able to absorb protons, thus acquiring a positive charge, which in turn allows its penetration into cells’ mitochondria. Such a mechanism accounts for the decrease in formation of the superoxide anion radical, due to mild decoupling of respiration and phosphorylation [17]. Theoretical modeling with density functional theory (DFT) corroborated this mechanism [12].

On the other hand, only *in vivo* studies can ultimately prove a concept of fullerene as an antioxidant agent and help develop new C_60_-based drugs. There are some novel biosensors allowing time-dependent experiments [18]. In the present paper, a simple yet efficient bacterial model based on *Escherichia coli* is utilized. Recently, it was used to develop a biosensor for detection of catechol [19] and to detect biofilm formation using localized surface plasmon resonance in real-time [20]. Here, we decided to apply an *E. coli* biosensor based on the MG1655 pKatG-lux strain, which is specific to one of the main reactive oxygen species—hydrogen peroxide (H_2_O_2_). The idea is to measure the biosensor response to the damaging agent (hydrogen peroxide) in the presence of fullerene solution in order to validate its anti- or prooxidant effect. Thus, in the current study, we report the effects of fullerene C_60_ on the vital activity of *E. coli*, in particular, biosensor strain *E. coli* MG1655 (pKatG-lux), in the presence of the hydrogen peroxide as an oxidative stress generator.

## 2. Materials and Methods

### 2.1. Preparation of Fullerene C_60_ Aqueous Solutions

Unmodified fullerene C_60_ bought from Sigma–Aldrich, St. Louis, USA, was prepared as follows: Aqueous solutions of fullerene C_60_ were prepared by sonication of TWEEN 80 solution 2% in deionized water with a Sonics Vibra-Cell VCX 130 ultrasonic dysmembrator at a power of 15 W by pulses of 3/3 s for 10 min. The effect of fullerenes in solutions at concentrations of 10^−15^ to 10^−2^ g/L was investigated. Solutions of TWEEN 80 2% and C_60_ in TWEEN 80 2% were autoclaved at 121 °C for 10 min.

Concentrations of solubilized C_60_ fullerenes were determined with a Beckman Coulter DU® 800 UV/Vis spectrophotometer by measuring the optical density of the supernatant. The molar extinction coefficient for fullerene C_60_ at 310 nm is ε_310 nm_ = 18900 L mol^−1^ cm^−1^. Initially, suspensions of C_60_ were prepared at the high concentration of 1 g/L, but after 15 min, the majority of the fullerene precipitated. Therefore, the stability of this solution was controlled. The initial concentration was 1 g/L (1.39 × 10^−3^ mol/L) and the supernatant was taken every day.

### 2.2. Measurement of the Fullerene Nanoparticles Size by Dynamic Light Scattering

Size of suspended C_60_ aggregates was determined by the dynamic light scattering [21] with Nano-flex (Microtrack) apparatus.

### 2.3. Bacterial Strain Used

The strain *E. coli* MG1655 (pKatG-lux) contains a hybrid plasmid into which the regulatory region (promoter and operator) is inserted, with the reporter gene lux-CDABE being isolated from the genomes of luminous bacteria. The PKatG promoter detects hydrogen peroxide down to a concentration of 5 × 10^−6^ mol/L, while the concentration of hydrogen peroxide causing the highest bioluminescence induction is equal to 10^−3^ mol/L. The lux AB genes encode the synthesis of luciferase subunits, lux-CDE reductase synthesis. The reductase is involved in the formation of the substrate luciferase—tetradecanal. Lucerase catalyzes the oxidation of tetradecanal with the emission of a photon of blue-green light (λ_max_ = 490 nm), which was detected by a luminometer. Integration time to record a response (significant bioluminescence increase) at such wavelength in the presence of H_2_O_2_ was equal to 30 min, which is in accordance with the range reported in literature: 5–20 min [22,23].

### 2.4. Bioluminescent Experiments

Antioxidant activity was determined with the help of bioluminescent tests as follows: The bioluminescent strain *E. coli* MG1655 pKatG-lux grown in a high-grade Luria–Bertani (LB) medium was used as a Lux-biosensor of hydroxide-anion radical [24]. Overnight growth was diluted into fresh LB to a 1 McFarland standard concentration, with the culture density being measured by DEN-1B densitometer "Biosan". The culture was then grown in a thermostat at 37 °C for 1.5 h, and finally, aliquots of the resulting culture (90 μL each) were transferred to sterile microplate wells. Solutions of TWEEN 80 (2%) and C_60_ were added to the control and the remaining cells, respectively, and then incubated for 30 min in a 96-well microplate in a thermostat at 37 ± 0.5 °C. After that, hydrogen peroxide at a concentration of 10^−3^ mol/L was added. For the control, we used cells with the addition of only TWEEN 80 (2%) to the culture. The microplate was placed in the Immunotech LM-01T microtiter plate luminometer, equipped with a thermostat to incubate it at 37 °C. The bioluminescence intensity was measured every 5 min.

Biological effects of fullerene C_60_ on the parameters of oxidative stress were examined using a bacterial biosensor with the pKatG promoter, with hydrogen peroxide at a concentration of 10^−3^ mol/L being used as an inducer of bioluminescence and an oxidative stress factor.

Solutions of fullerene C_60_ were added 30 min before conducting the test. Hydrogen peroxide concentration was equal to 10^−3^ mol/L.

### 2.5. Calculation of the Protective Effect

Bioluminescence induction factor *I* was calculated relative to the control cells using standard procedure, as described in [23,25,26], i.e., as *I = (L_e_ – L_c_)/L*_c_, where *L*_e_ – *L*_c_ are luminescence intensities of the experimental and control specimens, respectively. Protective activity was calculated similarly as *P = (*1 *- I_f_/I_c_) ×* 100%, where *I*_f_ and *I*_c_ are induction factors of the solution with and without added fullerene, respectively.

## 3. Results

### 3.1. Stability of the Aqueous Solution. Determination of C_60_ Concentration

Stability of the aqueous solution is shown in Table 1.

Table 1 indicates that C_60_ in the supernatant remains at the concentration of the order of 10^−2^ g/L. Based on these results, further studies of the biological properties of C_60_ were carried out, utilizing more stable solutions with an initial fullerene concentration of 0.01 g/L (1.39 × 10^−5^ mol/L). According to spectrophotometric findings, it approximately corresponds to the saturation concentration for C_60_ in a 2% aqueous solution of TWEEN 80.

Optical density of the fullerene suspension supernatant was equal to 0.29 arbitrary units, which corresponds to a molar concentration of 1.54 × 10^−5^ mol/L or 0.0111 g/L.

### 3.2. Size of C_60_ Fullerene Nanoparticles in Solution

**Figure 1:**
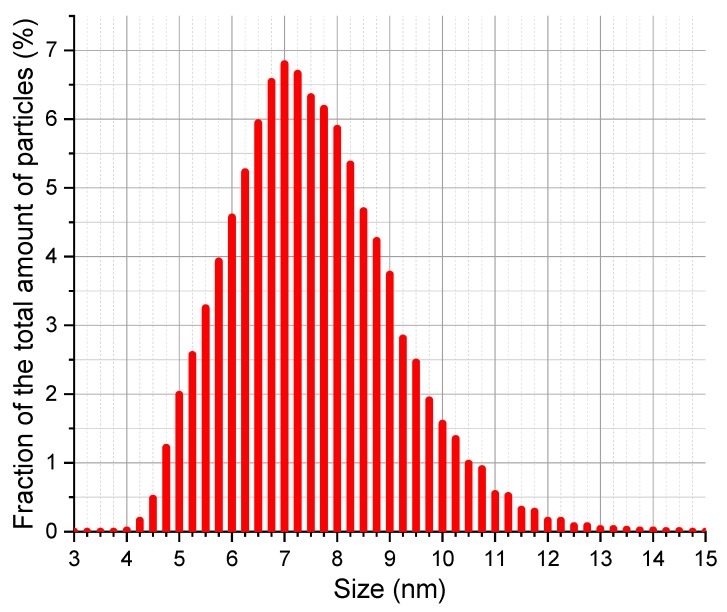
Size distribution of fullerene C_60_ in 2% solution of TWEEN 80 in water.

Particle size measurements are shown in Figure 1. The histogram demonstrates how the majority of aggregates (94.4%) vary in size in the range of 4 to 10 nm, with most (54%) of the C_60_ aggregates having a size from 6 to 8 nm; only 3.9% of aggregates have smaller diameters up to 5 nm. Resulting mean size is approximately 2–4 times larger than supernatant aggregates obtained in [27]. It was necessary to make sure that the deviation in cluster sizes was small enough because fullerenes can have different properties, e.g., in [27], larger aggregate size was found to be associated with lower antibacterial activity. Besides, according to [28], size of C_60_ aggregates and solution concentration may be dependent.

### 3.3. Biological Effects of Fullerene C_60_

**Figure 2:**
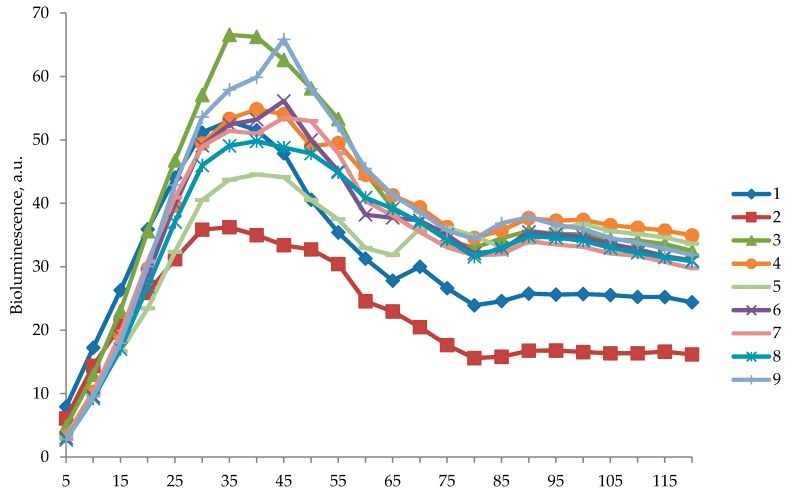
Bioluminescence induction factor of *Escherichia coli* strain MG1655 (pKatG) in the presence of hydrogen peroxide (H_2_O_2_) in the concentration of 10^−3^ mol/L, with an addition (within 30 min) of C_60_ suspensions in 2% aqueous solution of TWEEN 80 in different concentrations, varying from 10^−15^ g/L to 10^−3^ g/L. 1: TWEEN 80 with hydrogen peroxide (H_2_O_2_); 2: H_2_O_2_; 3: Fullerene C_60_ in a concentration of 10^−15^ g/L with H_2_O_2_; 4: C_60_ 10^−12^ g/L with H_2_O_2_; 5: C_60_ 10^−9^ g/L with H_2_O_2_; 6: C_60_ 10^−6^ g/L with H_2_O_2_; 7: C_60_ 10^−5^ g/L with H_2_O_2_; 8: C_60_ 10^−4^ g/L with H_2_O_2_; 9: C_60_ 10^−3^ g/L with H_2_O_2_.

**Figure 3:**
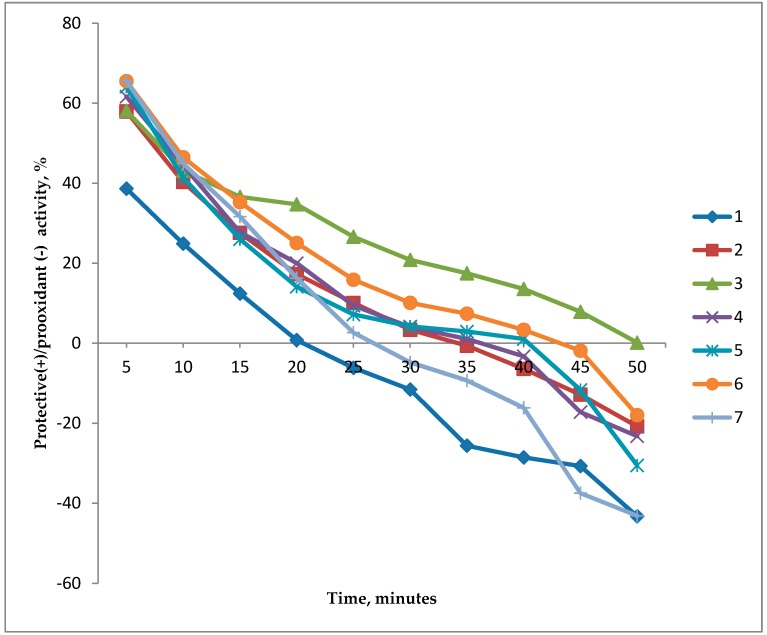
Comparative dynamics of the protective and prooxidant effects of C_60_, measured between 5 and 50 min after beginning of the experiment, %. 1: Fullerene C_60_ in a concentration of 10^−15^ g/L; 2: C_60_ 10^−12^ g/L; 3: C_60_ 10^−9^ g/L; 4: C_60_ 10^−6^ g/L; 5: C_60_ 10^−5^ g/L; 6: C_60_ 10^−4^ g/L; 7: C_60_ 10^−3^ g/L.

**Figure 4:**
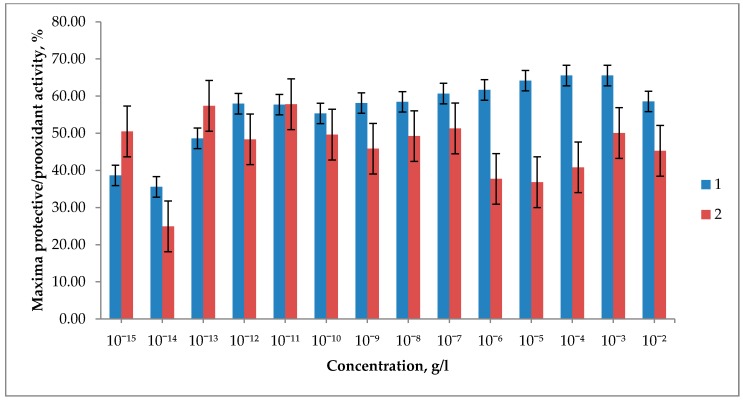
Maximum protective (1) and prooxidant (2) effect of the C_60_ solution in TWEEN 80 2% of different concentrations.

The bioluminescence induction factor for several tested concentrations is shown in Figure 2. Competition between the calculated protective and prooxidant effects is illustrated in Figure 3 and Figure 4. At all concentrations of C_60_ dissolved in 2% aqueous solution of TWEEN 80, the protective effect was first observed 15 min after beginning of the experiment, with a concentration of 10^−9^ g/L (1.39 × 10^−12^ mol/L) being an outlier with a prolonged time up to the minute 50. After 50 min, solutions of all tested C_60_ concentrations demonstrated a prooxidant effect (Figure 3).

In order to determine whether there is a relationship between the magnitudes of the highest values of the protective and prooxidant effects (Figure 4), the Pearson correlation coefficient between them was calculated. It equals 0.18, which, taking into account the small amount of degrees of freedom, means there is no statistically significant correlation between the maxima of the protective and prooxidant effect.

The most intensive protective effect was observed at C_60_ concentrations of 10^−3^ and 10^−4^ g/L, followed by 10^−5^ g/L (Figure 4). It can be explained by the concentration dependence of the cluster size distribution function in the C_60_ fullerene solution [28]. It implies that, under equal conditions, preparation of solutions at concentrations of 100 and 1000 times less than the saturation concentration significantly increases the share of small (up to 5 nm) clusters in comparison with the saturated fullerene solution.

**Figure 5:**
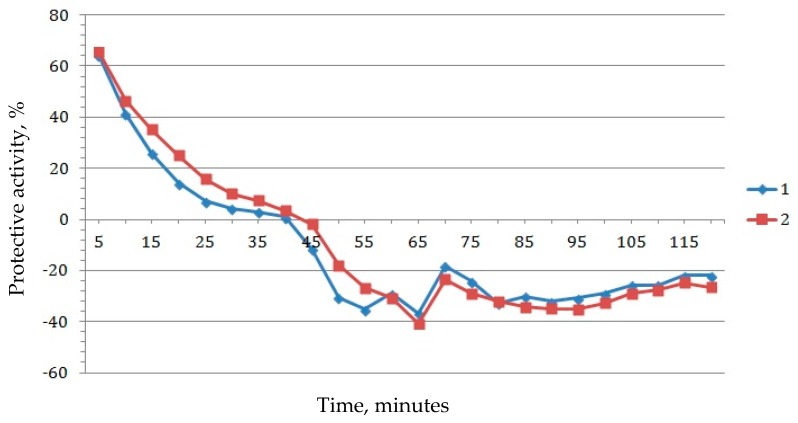
Protective effect time dependency for C_60_ in TWEEN 80 2% at concentrations 10^−5^ (1) and 10^−4^ (2) g/L.

**Figure 6:**
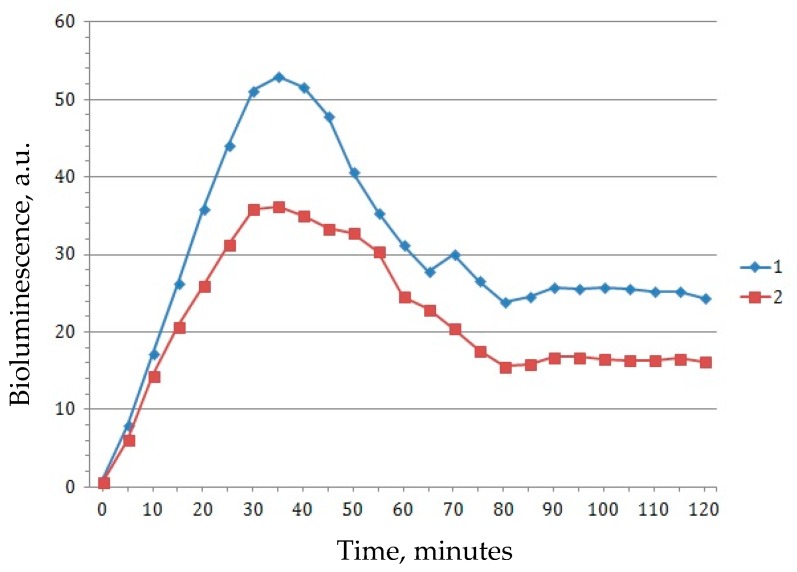
Increase of luminescence induction by hydrogen peroxide with and without the preliminary addition of TWEEN 80 2%. 1: Induction of luminescence by hydrogen peroxide in cells in which TWEEN 80 was previously added 2%; 2: Induction of luminescence by hydrogen peroxide without TWEEN 80.

Aqueous solutions of C_60_ were found to have both protective (antioxidant) and prooxidant effects, with the solution properties having changed at 20–50 min after the beginning of the experiment (Figure 3 and Figure 5). This may be due to a significant increase in the number of fullerenols formed from fullerenes, as compared to the amount of unmodified C_60_, as evidenced by the observation that the transition of the antioxidant effect to the prooxidant one occurs faster at lower concentrations (10^−15^–10^−13^ g/L) than at higher ones. In addition, it is worth noting that the induction of luminescence by hydrogen peroxide was significantly higher in the cells with TWEEN 80 as compared to the cells in which only peroxide was added (Figure 6).

One can conclude that it is necessary to take into account the aftereffects of using surfactants and other methods of obtaining nanoparticle solutions when studying their biological properties. It is important not only because chemicals used during preparation affect properties of nanoparticles in solutions, but also because they have influence on other compounds used in studies; in particular, on the interaction of cells with reactive oxygen species (ROS).

## 4. Discussion

Obtained results indicate that 50 min after the beginning of the experiment, most of the C_60_ molecules hydroxylated, due to cleavage of double bonds by hydrogen peroxide with the addition of OH groups, which changed biological properties in such a way, that the protective effect of fullerene turned into the prooxidant effect of fullerenol. Consequently, one should take this factor into account when studying biological properties of fullerene solutions.

There is, however, no direct linear relationship between the fullerene concentration and duration/intensity of the biological effect of the particular solution, which impedes derivation of the simple model relating C_60_ uptake kinetics and the oxidative response. In other words, there are many complicated factors playing a role in the *E. coli* response and are worth further investigation.

The biosensor *Escherichia coli* used in our study is a gram-negative bacillus bacterium, widely used as a model organism in microbiological studies. *E. coli* belongs to the same type (Proteobacteria) as the supposed ancestor of mitochondria and has similar dimensions. Moreover, *E. coli* has an external and internal membrane and (importantly for toxicological tests) its own antioxidant system with antioxidant enzymes similar to mitochondria, which justifies using this kind of bacteria as a mitochondria model.

Treating *E. coli* as an appropriate model of mitochondria, one can assume that fullerene C_60_ is able to act as a mitochondria-directed antioxidant. It is shown [29] that such structures in cell cultures disable H_2_O_2_-induced apoptosis; increase the lifespan of fungi and insects *in vivo*; suppress many signs of aging (geroprotective effect); reduce the formation of ROS in the mitochondria; and inhibit mitochondrial-mediated apoptosis, which is an obligatory step in the implementation of the program responsible for aging and rapid "biochemical suicide" after a severe metabolic crisis. If fullerene C_60_ is able to penetrate the mitochondria according to the mechanism proposed in [30], then it could become the basis for a new type of drugs.

In order to justify the extrapolation of the obtained results on mitochondria of eukaryotes, experiments on the eukaryotic organisms like nematodes are currently under way.

Buckminsterfullerene and its derivatives can be used as antioxidants, geroprotectors, drug delivery, and photodynamic therapy agents, provided the above-mentioned peculiarities are taken into consideration. Of particular importance is the complex behavior of fullerenes found in aqueous solutions and the adverse effects of the solvent as well as sizes of the fullerene aggregates and intense light, especially in the UV spectrum.

## 5. Conclusions

All tested concentrations of C_60_ in 2% aqueous solution of TWEEN 80 demonstrated an antioxidant effect during the first 15 min from the experiment start, while the concentration of 10^−9^ g/L demonstrated the effect even up to the 50th minute. A prooxidant effect was observed for all tested C_60_ concentrations after the 50th minute, which may be explained by the significant increase in the number of fullerenols formed from fullerenes as compared to the amount of unmodified C_60_. This statement is confirmed by a relatively earlier transition of the antioxidant effect to the prooxidant one at lower concentrations (10^−15^ and 10^−13^ g/L) in comparison with higher fullerene concentrations. 

Moreover, the surfactant used for the dissolution of nanoparticles was found to have a significant influence on the enhancement of the luminescence of a bacterial biosensor when using a standard inducer—hydrogen peroxide.

## Figures and Tables

**Table 1:** Dynamics of stability of 2% aqueous solution of C_60_ in TWEEN 80.

Day	10 min Stirring, Followed by Ultrasound 15 W			
		Absorbance (arb. Units)	C_M_, mol/L	C, g/L
1		1.86	9.82 × 10^−^^5^	7.07 × 10^−^^2^
2		1.62	8.56 × 10^−^^5^	6.16 × 10^−^^2^
3		1.60	8.46 × 10^−^^5^	6.09 × 10^−^^2^
